# Initiation of immunoglobulin therapy by subcutaneous administration in immunodeficiency patients naive to replacement therapy

**DOI:** 10.1186/s13223-014-0063-8

**Published:** 2014-12-06

**Authors:** Alan P Koterba, Mark R Stein

**Affiliations:** Allergy Associates of the Palm Beaches, 840 US Highway 1, Suite 235, North Palm Beach, FL 33408-3340 USA

**Keywords:** Globulins, Immune, Immunoglobulins, Subcutaneous, Immunoglobulins, Intravenous, Immunoglobulin therapy, Immunological deficiency syndromes

## Abstract

**Background:**

Patients with immunodeficiency diseases require lifelong treatment with immunoglobulin (Ig), yet few studies have vetted dosing strategies and effectiveness of Ig in older patient populations. Patients requiring subcutaneous (SC) Ig (SCIG) typically start with intravenous dosing before transitioning to SCIG weekly maintenance. In this retrospective review, we investigated an alternate strategy with higher initial SC doses among an older patient population with antibody deficiency syndromes.

**Findings:**

Records of 13 patients (mean age, 70 years) with antibody deficiencies who were naive to treatment with Ig were assessed. SCIG (Vivaglobin® [Immune Globulin Subcutaneous (Human), 16% Liquid] or Hizentra® [Immune Globulin Subcutaneous (Human), 20% Liquid]) was given twice weekly (100 mg/kg) for 2 weeks, followed by weekly (100 mg/kg) administration The mean pretreatment IgG level was 460 mg/dL; at 1, 3, and 6 months after SCIG initiation, mean IgG serum levels were 852, 907, and 943 mg/dL, respectively. Maintenance doses were unchanged during 6 months of follow-up. All patients remain on SCIG (median, 44 months). One patient developed sepsis/cholangitis unrelated to treatment 3 months after starting SCIG; no other serious bacterial infections were reported.

**Conclusions:**

Initiation of SCIG by doubling the maintenance dose over 2 weeks may be a well-tolerated and effective option for patients with antibody deficiencies requiring Ig replacement, especially among older patients.

## Background

Primary immunodeficiency diseases (PIDDs) that arise from defects in immunoglobulin (Ig) function or production are chronic conditions that predispose patients to repeated infections, primarily bacterial in origin [1,2]. Patients with these types of PIDDs require lifelong treatment with Ig replacement therapy administered via the intravenous (IV) or subcutaneous (SC) route [3]. Secondary immunodeficiencies (SIDs) are also common in older patients because of lymphoproliferative disorders or as a result of chemotherapy, the use of corticosteroids, or immunosuppressive treatments [4]. These patients also lack IgG and antibody response to immunization and require treatment with IV Ig (IVIG) or SC Ig (SCIG) [4]. Both IVIG and SCIG are considered safe and have similar efficacy profiles [5]. In some elderly patients, however, the presence of comorbidities, including pre-existing cardiovascular disease, renal insufficiency, or hyperosmolarity, may contraindicate the use of IVIG therapy [6].

The prescribing information for SCIG products approved by the United States Food and Drug Administration and Health Canada notes that SCIG therapy should be initiated one week after the last IVIG dose [7-10], the use of which should have been ongoing for at least 3 months [7]. Limited guidance is available with which to evaluate the optimal administration schedule for initiating therapy with SCIG. Direct initiation with a 16% SCIG product was shown to be safe in a prospective, open-label, multicenter, 6-month study in 18 patients naive to Ig replacement therapy (patients were newly diagnosed) [11]. SCIG was initially administered at 100 mg/kg for 5 consecutive days, followed by maintenance dosing at 100 mg/kg per week. This regimen resulted in stable IgG levels and protection against infection [11].

Elderly patients (aged ≥65 years) constituted approximately 9% of the population with PIDD in the United States in 2007, which represented an increase compared with past years (1996/1997 [5%] and 2002 [4%]) [12]. Older patients with PIDD have a higher rate of comorbid serious, chronic disease than those with PIDD who are aged ≤64 years [12].

In this retrospective case review, the safety and efficacy of initiating IgG therapy with the SCIG products Vivaglobin® (Immune Globulin Subcutaneous [Human], 16% Liquid) and Hizentra® (Immune Globulin Subcutaneous [Human], 20% Liquid [both CSL Behring, LLC, King of Prussia, PA]) were assessed in older patients with PIDD or SID without either prior or recent IVIG treatment.

## Methods

The charts of patients from a single practice who had been diagnosed with PIDD (as defined by hypogammaglobulinemia and a lack of adequate response to pneumococcal or other vaccinations) and who received Ig replacement therapy between March 2007 and July 2012 were retrospectively reviewed. Two patients were diagnosed with SID with a known prior history of non-Hodgkin lymphoma. Patients without a prior or recent (within 6 months) history of IVIG use before initiation of SCIG were selected. Therapy was initiated with SCIG (100 mg/kg) twice weekly for 2 consecutive weeks and then weekly thereafter at the same total dose. This retrospective review met the conditions for institutional review board exemption under 45 CFR 46.101(b)(4). Two of the patients were included in a previous publication regarding SCIG therapy in elderly patients [13].

The total initial SCIG dose was based on standard IVIG loading doses. The decision to split the initial dose into 4 infusions over a 2-week period was based on patient convenience and local tolerability considerations. Patients received the initial SCIG dose in their physician’s office, with instructions on home-based administration. If patients required further assistance, a specialty pharmacy nurse visited the patient at home. Patients received subsequent SCIG doses, including the remaining initiation doses, at home.

Serum Ig levels were measured at baseline and at 1, 3, and 6 months following the start of SCIG treatment, which is the standard interval for IgG assessments in our clinical practice and was not part of a separate protocol. Serious bacterial infections (SBIs) and local injection-site reactions were assessed by reviewing patient charts for interval office visits and any notes or comments regarding the occurrence of an SBI during SCIG administration were recorded.

## Results

Thirteen patients (mean age, 70.2 years) without recent or prior IVIG therapy were identified as having had treatment initiated directly with SCIG therapy. All of the patients had hypogammaglobulinemia; 11 patients had common variable immunodeficiency (CVID) or hypogammaglobulinemia with specific antibody deficiency (Table 1) had SID related to a history of non-Hodgkin lymphoma (although meeting the criteria for CVID with low immunoglobulin levels, recurrent sinopulmonary infections, and poor post-pneumococcal vaccination response). One patient had experienced a hemorrhagic stroke (unrelated to IVIG) while on prior IVIG therapy and had not received IVIG treatment for >6 months; 2 patients began SCIG therapy de novo because of contraindications to IVIG therapy; and the remaining patients began SCIG therapy de novo because of convenience or insurance issues.

**Table 1:** **Background characteristics of patients treated with SCIG**

**Characteristic**	
Total, n	13
Mean age, years (range)	70.2 (61 − 83)
Sex	
Male, n	6
Female, n	7
Race, white,%	100
Mean (SD) baseline serum IgG, mg/dL	460 (145.9)

IgG = immunoglobulin G; SCIG = subcutaneous immunoglobulin; SD = standard deviation.

Ten patients initially received Vivaglobin; 3 patients received Hizentra. Patients who initially received Vivaglobin switched to treatment with Hizentra because of the discontinuation of Vivaglobin from the market. The median duration of treatment with Vivaglobin was 23.5 months (range, 11–33 months). No dose adjustments were made when the switch was made to treatment with Hizentra, except for 1 patient who was administered a split dose, twice weekly. The median duration of all SCIG therapy (including the initial dose) was 44 months (range, 32–60 months). Patients used 2 to 4 sites for each infusion. During the time of this retrospective review, total weekly doses were not modified for any patient.

**Figure 1:**
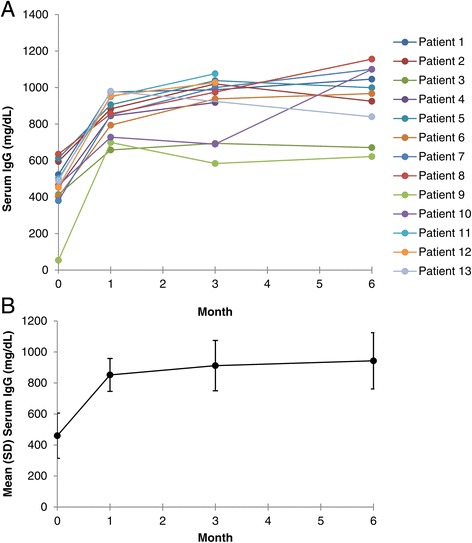
**Serum IgG levels in A) individual patients and B) mean of patients treated with SCIG.** Patients 1 − 10 began treatment with Vivaglobin and later changed to Hizentra. Patients 11 − 13 began and continued treatment with Hizentra. **B)** Mean serum IgG levels in patients treated with SCIG. Error bars indicate standard deviation. IgG = immunoglobulin G; SCIG = subcutaneous immunoglobulin.

Baseline IgG levels were recorded from <1 month to up to almost 8 months before the first SCIG administration. Serum IgG levels increased compared with baseline for all patients (Figure 1A). The mean ± SD baseline IgG level was 460 ± 146 mg/dL. The mean ± SD serum IgG level at month 1 rose to 852 ± 106 mg/dL (n = 13 patients); levels were 907 ± 157 mg/dL at month 3 (n = 12 patients) and 943 ± 182 mg/dL at month 6 (n = 10 patients; Figure 1B). Serum IgG levels were not available from 1 patient at 3 months and from 3 patients at 6 months.

Initial doses of SCIG and doses of weekly SCIG 100 mg/kg were well tolerated. No patients reported systemic adverse events and no patients discontinued SCIG treatment because of adverse events. Local site reactions, assessed subjectively by the attending physician, were mild in 46% of patients (6/13) and moderate in 15% of patients (2/13); 38% of patients (5/13) reported no local site reactions.

Only 1 SBI was reported during the entire SCIG treatment period; sepsis/cholangitis, thought to have been triggered by a gallstone, occurred in 1 patient approximately 3 months after the initial dose of Vivaglobin and was considered to be unrelated to PIDD. The patient’s SCIG dose was not changed during the infection; however, the patient did not receive SCIG during hospitalization because of elevated transaminases unrelated to PIDD.

## Discussion

Patients with PIDD or SID and without prior or recent IVIG therapy were effectively and safely treated with Vivaglobin® (16% SCIG) or Hizentra® (SCIG 20%) using an initial 2-week SCIG period, followed by weekly maintenance treatment with SCIG. IgG replacement therapy is generally initiated with doses ranging from 400 to 600 mg/kg/month in patients with PIDD, although higher doses may be required in patients with agammaglobulinemia or severe hypogammaglobulinemia [3]. Prescribing information for SCIG recommends that the dosage should be based on prior IVIG dosage [7-10]. However, as the use of SCIG increases, it would be reasonable for patients who require or desire IgG treatment with SCIG to initiate therapy with SCIG rather than IVIG. Despite differing initial regimens, both this retrospective review and a previously published trial [11] demonstrated that by 1 month after SCIG initiation, IgG levels were approximately double those at baseline. In the previous trial, mean serum IgG levels increased from 360 mg/dL at baseline to 740 mg/dL at day 26 [11]. In both reports, levels appeared to continue to rise slightly over the next few months, but a steady-state IgG level was reached within at least one month.

Infection rates appeared to be low in both reports: rates in the previous study [11] were reduced compared with the untreated period. Only 1 SBI, which was considered unrelated to treatment, was noted (approximately 3 months after the first dose) in the 44-month median follow-up in this retrospective review. Thus, these reports indicate that initiating Ig therapy with an SCIG dosing regimen can generate adequate IgG levels in patients with hypogammaglobulinemia that may be protective against serious systemic infections within at least 1 month.

The patients in this retrospective review were older, ranging from 61 to 83 years of age (77% of patients were aged ≥65 years). SCIG administration may be especially important in older patients for several reasons, including venous access and flexibility in scheduling. Obtaining IV access is often difficult in these patients, and some may require transportation to specialized facilities, rather than standard office settings, to receive infusions. Previous clinical assessments of Vivaglobin included few older patients [14], although a retrospective chart review of 47 elderly patients receiving SCIG (with no details of the loading regimen) showed therapy to be safe and effective [13]. No new safety concerns were identified in the older patients in this retrospective review. Injection-site reactions, expected with SC treatment [11], were mild or moderate when they occurred, and none led to discontinuation of treatment. Although not directly assessed in this retrospective review, prior studies have shown that the ability to administer IgG using the SC route, especially in a home-based setting, confers advantages in terms of health-related quality of life [15-17]. This respective review involved a small number of patients, and its open-label nature limits the strength of conclusions that can be drawn. Based on these promising preliminary results, use of a SCIG dosing regimen for initiation and maintenance of Ig therapy appears effective and generally well tolerated in older patients with PIDD.

## Abbreviations

CVID: Common variable immunodeficiency
Ig: Immunoglobulin
IV: Intravenous
IVIG: Intravenous immunoglobulin
PIDD: Primary immunodeficiency disease
SBI: Serious bacterial infection
SC: Subcutaneous
SCIG: Subcutaneous immunoglobulin
SID: Secondary immunodeficiency
